# Filarial Epididymitis: A Diagnostic Challenge in Pediatric Acute Scrotum

**DOI:** 10.7759/cureus.74534

**Published:** 2024-11-26

**Authors:** Jithu Thankachan, Neeraj V Mohandas, Venugopal PR, Akhila Lakshmi R S, Meghna Biji Philip

**Affiliations:** 1 General Surgery, Mount Zion Medical College Hospital, Adoor, IND; 2 Community Medicine, Dhanalakshmi Srinivasan Medical College and Hospital, Perambalur, IND

**Keywords:** acute scrotum, filarial epididymitis, filariasis, paediatric surgery, public health

## Abstract

Genital lymphatic filariasis (LF) is a condition that can present both acutely and chronically, complicating its diagnosis due to nonspecific symptoms. This case report describes an 11-year-old boy who presented with symptoms of acute scrotum. Initial Doppler ultrasound suggested acute epididymo-orchitis; however, despite conservative management, the symptoms persisted, prompting surgical exploration. The surgery revealed an edematous epididymis with a cystic lesion, leading to a diagnosis of filarial epididymitis. This case highlights the diagnostic challenges associated with genital filariasis and underscores the importance of considering it in the differential diagnosis of scrotal swelling, especially in endemic regions. Surgical intervention not only confirms the diagnosis but also alleviates symptoms and helps prevent future complications such as infertility.

## Introduction

Lymphatic filariasis (LF) is a debilitating disease often contracted in childhood and is among the leading causes of global disability [1]. Developing countries, such as India, Indonesia, Nigeria, and Bangladesh, bear the brunt of this disease, accounting for approximately 70% of global cases, according to the WHO [2]. The infection is caused by the nematode worms *Wuchereria bancrofti* or *Brugia malayi*, which are transmitted by the mosquito species *Culex quinquefasciatus *and *Mansonia *species, respectively [1]. *W. bancrofti *is responsible for nearly 90% of the disease burden, while *B. malayi *accounts for the remaining 10% [3].

LF affects over 120 million people across 73 countries [4]. The clinical presentation of *W. bancrofti *filariasis varies significantly depending on the infection’s stage and duration [5]. Genital LF, one of the disease’s manifestations, involves inflammation of the scrotal structures, leading to acute epididymo-orchitis or funiculitis in bancroftian filariasis. This condition is characterized by severe pain, tenderness, and swelling of the scrotum, often accompanied by fever and chills. The testes, epididymis, or spermatic cord may become enlarged and extremely sensitive, with secondary infections potentially exacerbating these symptoms [2]. USG can identify and visualize the movements of live adult *W. bancrofti *filarial worms in the scrotal lymphatics of asymptomatic males with microfilaremia [3,6]. The thrashing movements of the adult worms in their “nests” within the scrotal lymphatics are referred to as the “filaria dance sign” [7]. The treatment of choice for filariasis is diethylcarbamazine (DEC), effective against both microfilariae and adult worms [3]. Surgical intervention is often required for managing genital LF [8].

The term “acute scrotum” refers to a constellation of symptoms, including new-onset pain, swelling, and/or tenderness of the intra-scrotal contents [9]. It encompasses a range of conditions, from benign and self-limiting to malignant and life-threatening emergencies. Timely and accurate diagnosis is essential to initiate appropriate management and prevent complications such as testicular ischemia, necrosis, or infertility. Although rare, genital LF can present with an acute scrotum. This case report describes an unusual presentation of filarial epididymitis in a young child who presented with symptoms of acute scrotum.

## Case presentation

An 11-year-old boy from Pathanamthitta district in the southern Indian state of Kerala presented to the outpatient department with a four-day history of left scrotal pain and diffuse lower abdominal pain radiating from the lower abdomen to the left scrotum, accompanied by low-grade intermittent fever. The patient had no symptoms of vomiting, abdominal distension, or dysuria. He had no significant medical history, no history of trauma, and no history of travel to filariasis-endemic regions. There was no significant family history of filariasis.

Examination findings

On examination, the abdomen was soft and non-tender. The right scrotum showed erythema and loss of rugosity, with an elevated right testis, local warmth, and tenderness. A soft, well-defined mass measuring approximately 10 mm × 12 mm was palpated. No inguinal lymphadenopathy was noted, and a urological examination ruled out hydrocele, varicocele, or other abnormalities. The general physical examination revealed no positive findings. Table 1 presents the CBC report of the patient at the time of admission.

**Table 1:** CBC report at the time of admission

CBC	Value	Reference
Hemoglobin	13.5 gm/dl	12-16 gm/dl
Total WBC count	12,670 cells/cu mm	4,000-11,000 cells/cu mm
Neutrophils	73%	40-70%
Lymphocytes	16%	25-40%
Eosinophils	9%	01-06%
Monocytes	2%	02-08%
Basophils	0%	0.5-1%
Platelet count	3.3 lakhs/cu mm	1.5-4 lakhs/cu mm
RBC count	4.77 million/cu mm	3.8-4.8 million/cu mm

**Figure 1:**
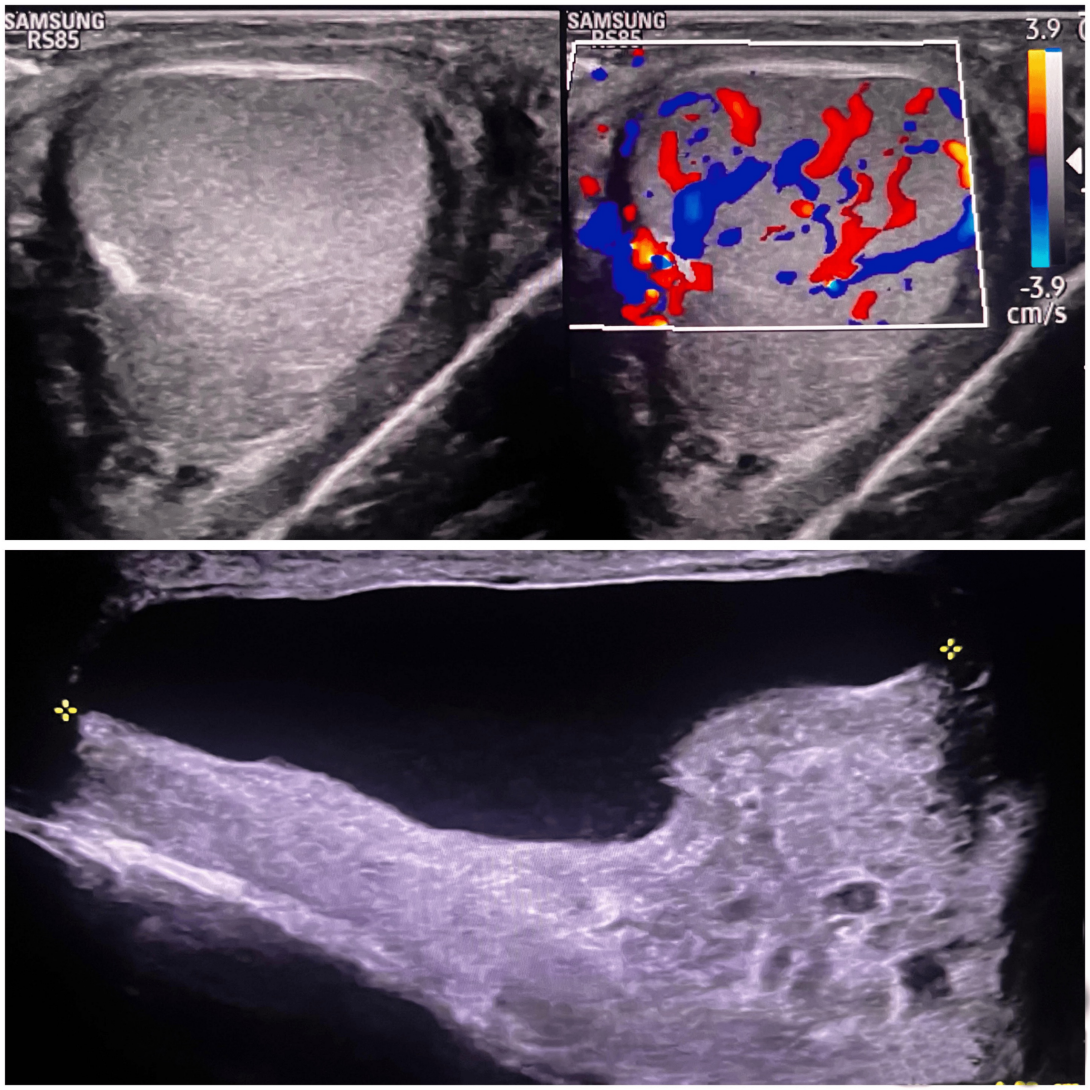
USG of the left scrotum (at the time of admission) showing features of epididymo-orchitis – increased vascularity and secondary hydrocele

**Figure 2:**
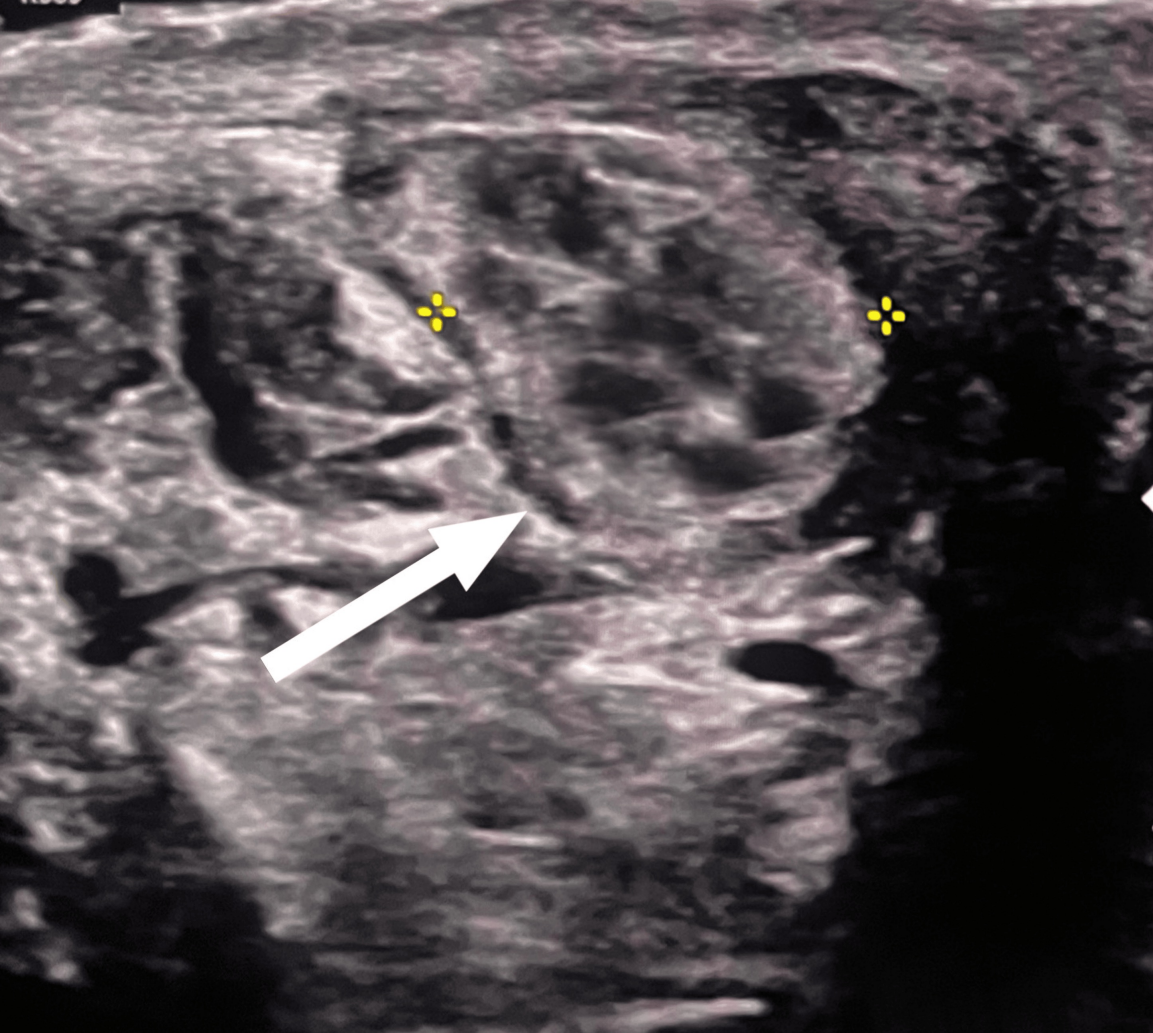
USG of the left scrotum showing a hypoechoic mass in the left epididymal head

Doppler USG performed at the time of admission indicated acute epididymo-orchitis (Figure 1). The patient was treated conservatively with oral doxycycline (100 mg twice a day) and analgesics for one week; however, the symptoms persisted. A follow-up ultrasound revealed free fluid in the tunica vaginalis with floating echogenic particles, along with a hypoechoic mass measuring approximately 11.9 mm × 7 mm at the upper pole of the left testis, showing normal vascularity and a whorled hyperechoic structure (Figure 2). Blood tests revealed elevated neutrophil and eosinophil counts (Table 1). The primary differential diagnoses included appendicular torsion and an infected epididymal cyst.

Surgical intervention

**Figure 3:**
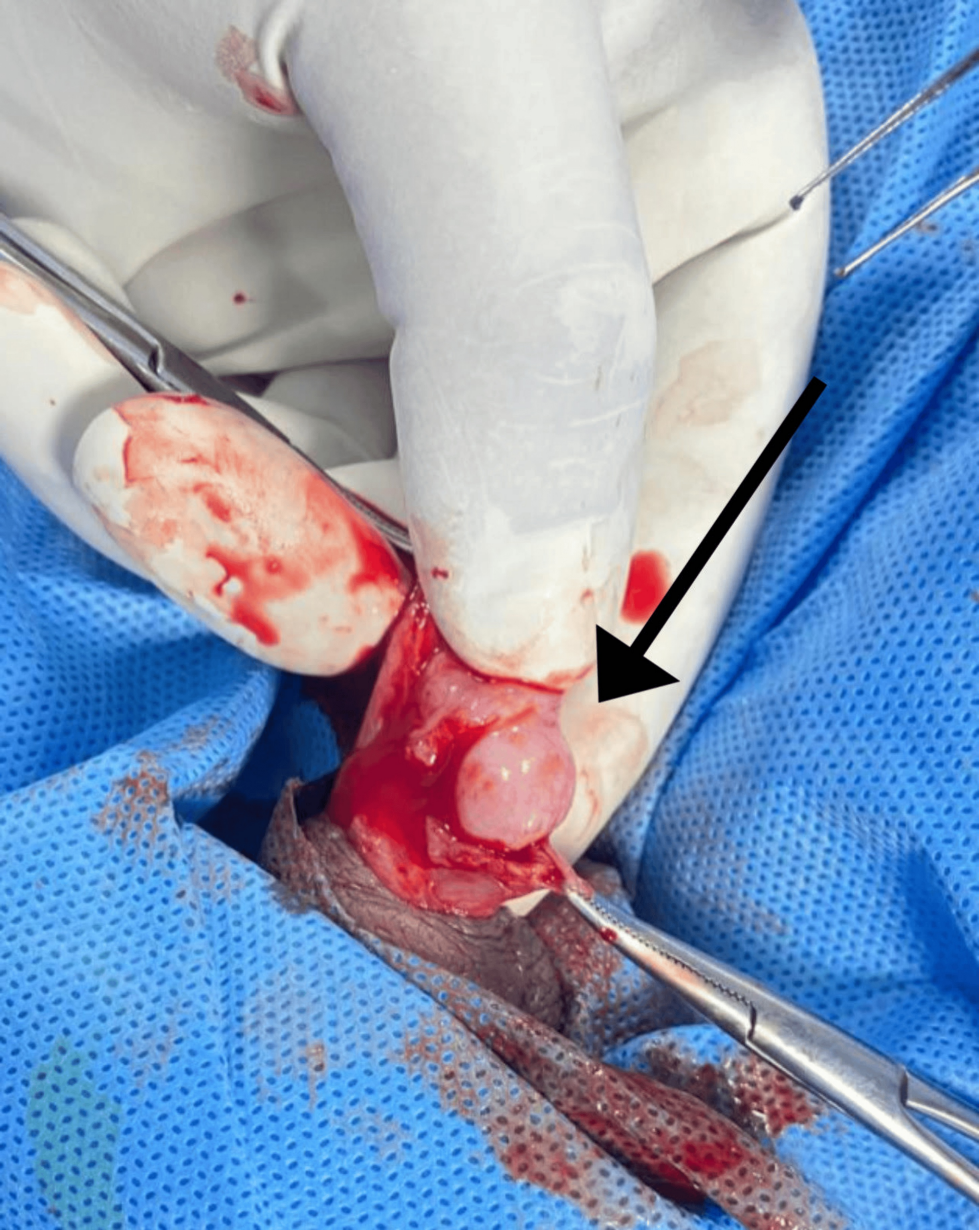
Surgical exploration of the left scrotum showing filarial cystic lesion with the epididymis

Owing to the persistence of symptoms, a surgical approach was considered. Scrotal exploration under general anesthesia revealed an edematous epididymis and a mass of approximately 10 mm × 8 mm located over the upper pole of the left testis (Figure 3). The mass was excised from both the left testis and the epididymis. Additionally, the hydrocele sac was excised, and a left orchidopexy was performed. There were no intraoperative or postoperative complications.

Histopathological findings

**Figure 4:**
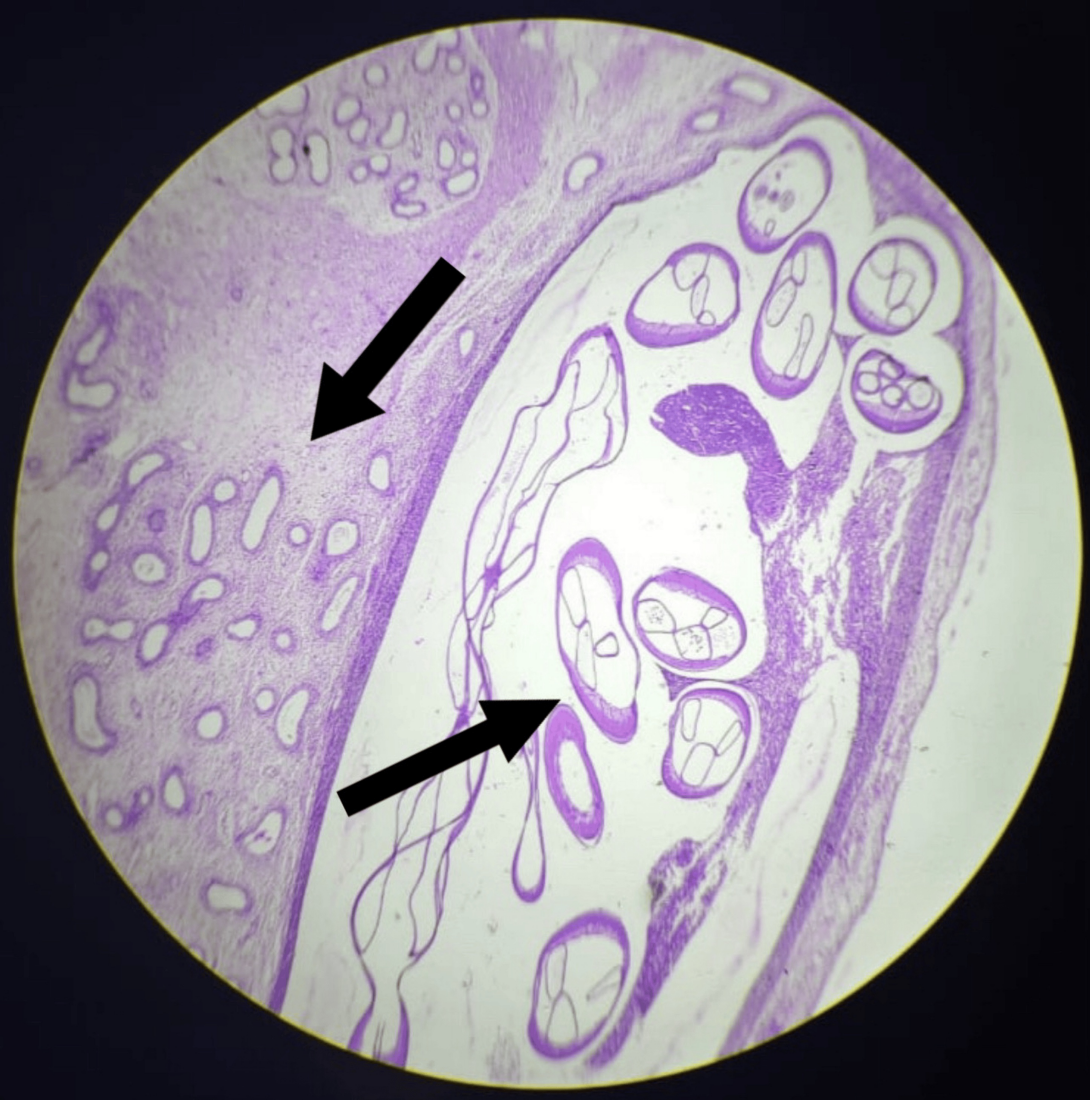
Microscopic slide showing seminiferous tubules and cross-section of the filarial worms (40x magnification)

**Figure 5:**
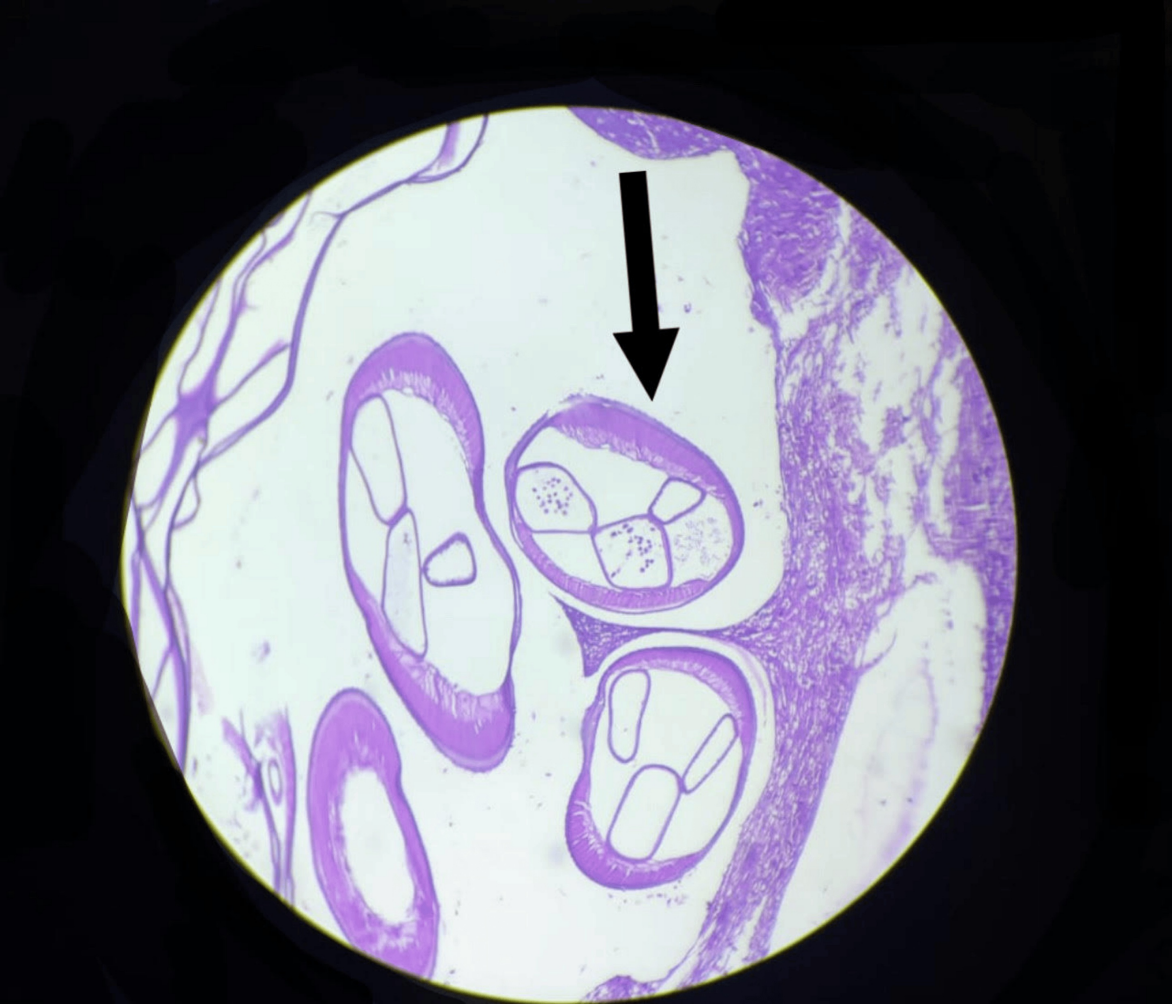
Microscopic slides showing the cross-section of the filarial worm with thick walls (400x magnification)

Histopathology revealed a fibrinous exudate and a cystic space containing cross-sections of a parasite with thick, chitinous walls, muscle bundles, and intestinal tubules (Figure 4, Figure 5). There was no evidence of malignancy.

## Discussion

The WHO reports that 50% of people infected with LF are from Southeast Asia [10-12]. India accounts for 40% of the world’s LF cases, with the majority of districts in the southern state of Kerala being endemic for filariasis [13]. The infection primarily affects the lymphatic channels, leading to dysfunction, obstruction, and inflammation. LF is extremely rare in non-endemic regions [11,14]. Genital filariasis can result in hydrocele, lymphatic varix, filarial penis, genital elephantiasis, and filarial epididymitis. Hydrocele with associated epididymo-orchitis is one of the most common presentations of genital filariasis, although the discovery of an adult worm in the epididymis is extremely rare. In non-endemic areas, such as in this case, the cause of genital filariasis may be attributed to mosquitoes - vectors of human filarial infection - originating from endemic regions [15].

Genital filariasis may present as either acute or chronic. Acute infection typically presents with epididymo-orchitis, along with milder symptoms and no systemic manifestations [6]. Chronic cases may present with symptoms due to permanent obstruction of lymphatic vessels, often resulting in a hydrocele of the tunica vaginalis [16]. As observed in our case report, diagnosing genital LF preoperatively is challenging due to its rarity, the lack of specific clinical signs, and the absence of characteristic findings. Notably, the “filarial dance sign” on ultrasound was absent in this case, and the role of fine needle aspiration cytology remains questionable due to the risk of dissemination, especially when malignancy is suspected [17]. While doxycycline is thought to be effective in managing the initial stages of filariasis, the treatment of choice for genital LF remains DEC [8]. In this case, doxycycline and analgesics were initially prescribed for a week; however, due to persistent symptoms, surgical exploration and histopathological examination were performed, confirming that the cause of the filarial epididymitis was genital LF.

Medical management is typically the first-line treatment for genital filariasis. Surgical intervention is considered when symptoms persist, especially when fibrotic or calcified nodules need to be removed. Urogenital surgery is regarded as an effective treatment by the WHO and plays a critical role in both diagnosis and preventing complications such as infertility in young patients [18].

The debilitating effects of LF, including lymphoedema, elephantiasis, and hydrocele, impose a significant public health burden. Eleven out of the 14 districts in Kerala, India, are endemic for LF [19]. Both medical and surgical interventions can significantly improve the quality of life for affected individuals. When performed correctly, surgical procedures can effectively address genital LF. Government initiatives, coupled with public health education, are essential for controlling this neglected tropical disease.

This case underscores the importance of considering genital filariasis in the differential diagnosis when scrotal swelling does not respond to conservative management, particularly in non-endemic regions or cases with unusual presentations. Surgical management not only aids in diagnosis but also provides symptomatic relief, potentially preventing long-term complications such as infertility in young patients.

## Conclusions

This case report highlights the diagnostic challenges of genital filariasis, particularly in non-endemic regions. The presentation of acute scrotal pain, followed by the finding of epididymo-orchitis unresponsive to conservative management, underscores the need for a high index of suspicion for conditions like filarial infection, especially in LF-endemic areas. Despite initial conservative treatment, surgical intervention became crucial for both diagnosis and management, emphasizing the importance of surgical exploration in symptomatic cases. Early recognition and appropriate treatment are essential to prevent long-term complications, such as infertility.
